# Patient expectations and satisfaction in hand surgery: A new assessment approach through a valid and reliable survey questionnaire

**DOI:** 10.1371/journal.pone.0279341

**Published:** 2022-12-20

**Authors:** Shin Woo Choi, Joo-Yul Bae, Young Ho Shin, Young Joo Jung, Ha Sung Park, Jae Kwang Kim

**Affiliations:** 1 Department of Orthopedic Surgery, Gangneung Asan Hospital, University of Ulsan College of Medicine, Gangneung, Korea; 2 Department of Orthopedic Surgery, Asan Medical Center, University of Ulsan College of Medicine, Seoul, Republic of Korea; PLOS ONE, UNITED KINGDOM

## Abstract

**Introduction:**

Assessing patient expectations in orthopaedic surgery has gained significant importance over time. However, there have been only a few studies on how to measure such expectations in hand surgery. Against the backdrop, the study was designed to develop a valid and reliable expectations survey for patients undergoing hand surgery and to identify the correlations between preoperative expectations and postoperative satisfaction.

**Materials and methods:**

This is a three-phase prospective cohort study. In the first phase of the study (146 patients), patient expectations were assessed while developing a draft questionnaire based on frequency and clinical relevance. In the second phase (154 patients newly included), test-retest reliability was measured to ensure test consistency. The Intraclass Correlation Coefficient (ICC) served as a basis for developing the final survey questionnaire. In the third phase, we followed up with patients, who completed the preoperative expectations survey, 3 months after surgery to assess the fulfillment of their expectations. The Pearson correlation method was used to measure the association between preoperative expectations and postoperative satisfaction.

**Results:**

In the first phase, 146 patients shared 406 different expectations, which were grouped into nine categories. Then, in the second phase, the final survey was populated by questionnaire items under respective category that have revealed strong test-retest reliability (ICC of 0.91). A significant positive correlation between patient expectations and satisfaction was observed (R = 0.181, *p* = 0.034).

**Conclusion:**

The survey was designed to offer a valid and reliable approach for the comprehensive assessment of patient expectations in hand surgery. The survey results show that patients with high expectations tend to be more satisfied with surgical outcomes. It is strongly believed that this approach would serve as a useful tool at a time when patient perspective is taken into account increasingly more in the clinical practice.

## Introduction

In recent years, assessing patient expectations in orthopaedic surgery has gained significant importance. With better understanding of patient expectations for a surgical procedure, it would be possible to decide which treatment type to choose while facilitating patient education by managing expectations through feedback [1,2]. When patients come to hand surgeons, they think that hand surgery would be just a simple procedure since the hand takes up a small part of the body [3]. This misperception is the reason why patient expectations need be managed properly by educating patients regarding treatment complexity.

The best way to assess patient expectations is to use reliable and validated tools. In a review article, Zywiel et al. described tools used for assessing patient expectations in orthopaedic surgery and concluded that using highly sophisticated tools was crucial in understanding what patients expect and how such expectations are correlated with surgical outcomes [4]. His research team found that a large number of unvalidated tools were used in several studies, thereby limiting research outcomes.

Reliable and validated tools for assessing patient expectations have been developed for surgeries performed to treat hip, knee, spine, shoulder and foot [5–10]. However, there is not any standardized toolset to measure patient expectations in hand surgery. Developing a standardized measurement approach is an essential prerequisite for investigating preoperative patient expectations.

The study aims to design a valid and reliable survey to assess patient expectations in hand surgery and to identify the relationship between patient preoperative expectations and postoperative satisfaction.

## Materials and methods

This is a three-phase prospective cohort study. The study protocol was reviewed and approved by our institutional review board.

### Phase I: Assessing patient expectations and drafting a survey questionnaire

#### Patient selection

For Phase I, our research team enrolled 173 patients scheduled for elective hand surgery, including traumatic cases, in 2014 and 2016. Patients were recruited if the subject was older than 18 and if informed consent was obtained. On the other hand, patients were excluded if the subject was to undergo either emergency surgery or minor procedures including implant removal and simple mass excision.

The diagnoses were made based on the 10th revision of the International Classification of Diseases (ICD-10). We grouped the patients into seven major diagnostic categories as per clinical relevance and frequency: (1) distal radius fracture; (2) wrist tendinopathies including De Quervains; (3) diseases of ulnar-sided wrist pain including ulnar impaction syndrome and triangular fibrocartilage complex injury; (4) first carpometacarpal arthritis; (5) trigger finger; (6) cubital tunnel syndrome; and (7) carpal tunnel syndrome. The rest of the diagnoses, such as finger osteoarthritis, wrist ganglion, Kienbock disease, scaphoid nonunion, mallet injury and Dupuytren’s disease, were grouped as “Other diagnoses”.

Patient enrollment discontinued when no new expectations were brought to attention. Of 173 patients, 23 subjects were excluded following surgery cancellation while four other patients were also excluded because of a concomitant disease in the same hand. Ultimately, 146 patients were selected for Phase I.

#### Assessing patient expectations

All the patients were interviewed in the outpatient clinic a few days ahead of surgery, and an open-ended question was asked as follows: “What are your expectations for this hand surgery?” The responses were written down verbatim. All interviews were conducted in person without a time limit.

A panel of orthopaedic surgeons used a standard technique for qualitative data collection to analyze the responses and categorize expectations accordingly. All open-ended responses were reviewed and coded as per each category.

#### Drafting a survey questionnaire

A survey questionnaire to investigate hand surgery expectations was drafted based on categories mentioned by ≥ 5% of respondents or categories that were deemed clinically relevant by a panel of three orthopaedic specialists at the hospital. Questionnaire items were written with terminology that could be easily understood by patients.

The responses were scored on a scale of 1 to 5, where 5 signifies “very important” whereas 1 means “not important”. For reference, respondents were given the option to choose “this does not apply to me” when the question was not related to their disease. Then, the final score was calculated by averaging, instead of adding up, all the points after excluding the responses of “this does not apply to me”.

### Phase II: Testing reliability and finalizing a survey questionnaire

#### Patient selection

185 patients scheduled to undergo hand surgery from March 2017 to February 2018 were newly recruited to determine the test-retest reliability of the draft survey questionnaire. Of 185 patients, 31 subjects were excluded following the cancellation of index surgery. Therefore, 154 patients responded to both the first and second drafts of the survey questionnaire. The patient inclusion and exclusion criteria were identical to those used in Phase I. The demographic characteristics and distribution of diagnoses in Phases I and II are provided in Table 1.

**Table 1:** Demographic characteristics and diagnoses in Phases I and II.

	Phase I (146)	Phase II (154)	*p* value
Age (SD)	48 (16.03)	51 (16.08)	0.080
Female	59 (40.4%)	83 (53.9%)	0.019*
Distal radius fracture	33 (22.6%)	9 (5.8%)	< 0.001*
Wrist tendinopathy	11 (7.5%)	14 (9.0%)	0.626
Ulnar-sided wrist pain	9 (6.1%)	11 (7.1%)	0.734
First CMC arthritis	20 (13.6%)	18 (11.7%)	0.601
Trigger finger	12 (8.2%)	13 (8.4%)	0.944
Carpal tunnel syndrome	28 (19.2%)	45 (29.2%)	0.045*
Cubital tunnel syndrome	10 (6.8%)	17 (11.0%)	0.205
Other diagnoses	23 (15.7%)	27 (17.5%)	0.679

SD, standard deviation; CMC, carpometacarpal.

*p < 0.05.

#### Test-retest reliability

Test-retest reliability was assessed by administering the drafted questionnaires to patients on two separate occasions. The first questionnaire was administered at the outpatient clinic on the very day when a decision to operate was made whereas the second was administered upon hospitalization ahead of the scheduled surgery.

The test-retest reliability was measured based on the Intraclass Correlation Coefficient (ICC) between the average scores of the first and second surveys and between the scores of each questionnaire item. The ICC of ≥ 0.7 is required at minimum to be deemed as acceptable [11].

#### Finalizing a survey questionnaire

The questionnaire items for the final survey were selected based on ICC and potential clinical relevance.

### Phase III: Analysis of preoperative expectations and postoperative satisfaction

With regard to 154 patients who were recruited for Phase II, their demographic and clinical characteristics, such as age, gender, marital status, active work status, hand dominance, history of antecedent injury, visual analogue scale (VAS) and disabilities of the arm, shoulder and hand (DASH), were investigated.

Additionally, we followed up with all the patients who completed the preoperative expectations survey 3 months after index surgery to assess the fulfillment of their expectations. Of 154 respondents in the preoperative survey, 138 patients (89.6%) completed a 3-month follow-up satisfaction survey. We asked patients each questionnaire item to assess their postoperative satisfaction measured against preoperative expectations. The responses were, then, scored on a scale of 1 to 5, where 5 points signify “very satisfied” whereas 1 point means “not satisfied at all”. Just as with the expectations score, the final satisfaction score was calculated by averaging all the scores.

### Statistical analysis

IBM SPSS Statistics V22.0 was used to perform all the statistical analyses (IBM, Armonk, NY) with *p* < 0.05 defined as statistically significant. Continuous variables were reported in terms of mean and standard deviation, which were compared by t-tests. Categorical variables were compared using the Pearson chi-square test. The test-retest reliability of the survey was measured based on the ICC (two-way mixed-effect model, absolute agreement). In addition, expectations and satisfaction scores were assessed according to demographic characteristics and functional scores (VAS and DASH scores) through multivariable regression analysis. Stepwise regression models were used for analysis with the critical *p* value of 0.10 as the threshold for retention. In all the regression models, β-coefficients were obtained to assess the magnitude of the effects, with 95% confidence intervals (CIs) for accurate evaluation. Finally, Pearson correlation analysis was used to evaluate the association between preoperative expectations and postoperative satisfaction.

## Results

### Phase I

#### Drafting a survey questionnaire

146 patients shared a total of 406 expectations (2.78 expectations per patient on average) and rated 80% of the expectations as very important. The panel of orthopaedic surgeons grouped these expectations based on frequency and clinical significance. To reduce the response burden, questions were narrowed down to 9 in the draft questionnaire by combing similar concepts together.

The most frequently mentioned expectation was “improve the ability to perform daily activities”, reported by 81% of the patients. This expectation includes the ability to self-maintain personal hygiene, to dress, to do household chores (laundry, dish-washing, cooking, etc.), to draw and to write. The second most frequently mentioned expectation was “relieve, reduce and eliminate pain”, reported by 74% of the respondents. The expectation item of “improve the ability to participate in sports and leisure activities” was mentioned by 65% of the patients, which includes weight training, climbing, soccer, basketball, swimming, cycling, baseball, judo, tennis, table tennis, golf and boxing.

### Phase II

#### Test-retesting reliability

The second survey was conducted 4.3 weeks after the first survey on average, which was administered in the hospital ahead of surgery. The test-retest reliability of the draft survey questionnaire was high in general as attested by the ICC of 0.910 (95% CI, 0.874–0.936; *p* < 0.001; See Table 2). All nine questionnaire items had the ICC of ≥ 0.7.

**Table 2:** Frequency and ICC of each expectation in the final survey.

Expectation item	Frequency (%)	ICC	95% CI
All		0.910	0.874–0.936
Relieve/reduce/eliminate pain	74	0.821	0.747–0.873
Participate in sports and leisure activities	65	0.858	0.799–0.899
Perform daily activities	81	0.729	0.617–0.808
Return to work	45	0.821	0.729–0.881
Minimize post-operative scars	23	0.828	0.757–0.878
Avoid post-operative sequelae or complications	10	0.828	0.758–0.878
Restore joint mobility	18	0.856	0.798–0.898
Increase emotional stability	8	0.825	0.754–0.876
Correct hand deformities	6	0.838	0.771–0.885

ICC, intraclass correlation coefficient; CI, confidence interval.

#### Finalizing a survey questionnaire to assess patient expectations

**Fig 1:**
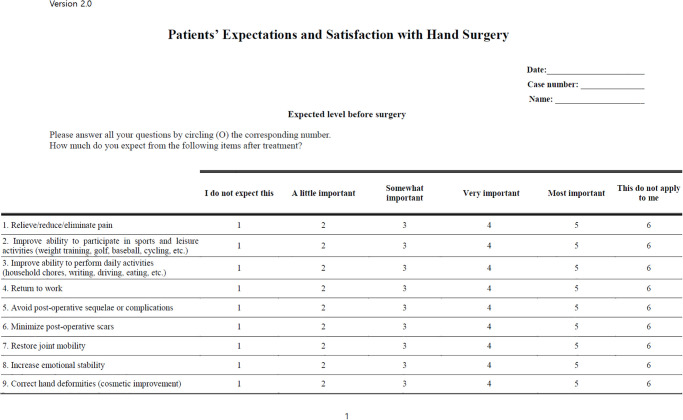
Survey of patient expectations in hand surgery.

Items in the draft questionnaire with the ICC > 0.7 were included in the final version. The clinical relevance of these nine items was also considered to develop the final questionnaire for investigating patient expectations in hand surgery (See Fig 1).

### Phase III

#### Analyzing preoperative expectations

Among the variables, female (β = 0.327; 95% CI, 0.063–0.591; *p* = 0.016) and age (β = 0.23; 95% CI, 0.015–0.030; *p* < 0.001) were found to be the patient factors showing significant association with higher expectations scores (See Table 3).

**Table 3:** Multivariable regression analysis for mean expectations score.

Variable	β-Coefficient	Standard error	95% CI	*p* value
Age	0.23	0.004	0.015–0.030	0.000
Female	0.327	0.132	0.063–0.591	0.016
Marital status	-	-	-	-
Active work status	-	-	-	-
Dominant side affected?	-	-	-	-
Prior hand injury history	-	-	-	-
Preoperative VAS	-	-	-	-
Preoperative DASH	-	-	-	-

CI, confidence interval; VAS, visual analogue scale; DASH, disabilities of the arm, shoulder, and hand.

#### Analyzing postoperative satisfaction

In the multivariable regression analysis of patient satisfaction, improvement in DASH (β = 0.27; 95% CI, 0.015–0.039; *p* < 0.001) and high preoperative expectations score (β = 0.323; 95% CI, 0.023–0.047; *p* = 0.023) were found to be the patient factors showing significant association with higher satisfactions scores (See Table 4).

**Table 4:** Multivariate regression analysis for mean satisfaction score.

Variable	β-coefficient	Standard error	95% CI	*p* value
Age	-	-	-	-
Female	-	-	-	-
Marital status	-	-	-	-
Active work status	-	-	-	-
Dominant side affected?	-	-	-	-
Prior hand injury history	-	-	-	-
Improvement in VAS	-	-	-	-
Improvement in DASH	0.27	0.006	0.015–0.039	0.000
High expectations score	0.323	0.138	0.023–0.047	0.023

CI, confidence interval; VAS, visual analogue scale; DASH, disabilities of the arm, shoulder, and hand.

#### Correlation between expectations and satisfaction

A significant positive correlation between patient expectations and satisfaction was observed (R = 0.181, *p* = 0.034).

## Discussion

With access to large patient sample, it was possible to design a valid and reliable approach for the assessment of patient expectations in hand surgery through nine questionnaire items derived from patients. The items included expectations related to symptoms, functions and aesthetic and psychosocial considerations. After the analysis of survey results, a positive relationship was identified between preoperative expectations and postoperative satisfaction.

Previous studies of patient expectations in hand surgery have been limited by specific conditions. A study of patients undergoing carpal tunnel release found that preoperative expectations did not affect patient outcomes, including satisfaction after treatment [12]. However, the study measured patient expectations based on their DASH scores, which is not a valid method. More recently, Kang et al designed a valid and reliable expectations survey for patients with thumb carpometacarpal arthritis [13] and reported that variable patient factors, including gender, were associated with greater preoperative expectations [14]. To the best of our knowledge, this study is the first of its kind to assess the expectations of patients undergoing surgeries for a broad-spectrum of hand diseases. As the expectations survey was designed based on heterogeneous diagnoses and disparate procedures, the survey questionnaire can be applied universally to hand patients in general.

A standardized methodology was used to design our expectations survey based on previous literature including Hospital for Special Surgery expectations surveys [5–10]. Content validity was established by interviewing 146 patients awaiting hand surgery and also by expert review of the responses. Test-retest reliability was also confirmed with another 154 patients who underwent hand surgery. In developing the final version of the survey questionnaire, items were simplified by combining similar concepts together instead of presenting items of specific nature separately. Our approach was consistent with that of other studies, which advocate keeping a survey simple and respondent-friendly to minimize response burden [15–18]. Similarly, the American Academy of Orthopaedic Surgeons developed the Musculoskeletal Outcomes Data Evaluation and Management System expectations scale with six-items, which are applicable to a wide range of musculoskeletal diseases [19]. The survey we have designed provides physicians with simple and clear representation of patient expectations in a validated and reliable method.

The expectation items of “minimize post-operative scars” and “avoid post-operative sequelae or complications” may be regarded as overlapping in some sense. However, the study found that these were mentioned as separate expectations by many respondents. Specially, the item of “avoid post-operative sequelae or complications” was the primary concern for patients worrying that something might go wrong during a surgery. It was regarded as a much more serious problem than post-operative scars. By contrast, the item of “minimize post-operative scars” strongly relates to the cosmetic aspect. Unlike other studies on patient expectations, several patients were concerned about “post-operative scars”. As scars on hands are more conspicuous to one-self and others, aesthetic considerations are important for some patients [20]. Since both items were based on what patients said in their own words, the wordings may not be as clear and medically precise as what physicians commonly use. Nevertheless, those wordings were adopted as they were in the survey because 23% and 10% of respondents respectively used those exact words to express their expectations in their open-ended interviews.

There are several studies reporting the effect of patient factors on preoperative expectations although majority of these studies have focused on arthroplasty, shoulder surgery and spine surgery. As a systematic review article of the current expectations research in orthopaedic surgery, Swarup et al. described that certain patient factors, including age and gender, have been found to influence preoperative expectations in total hip and total knee arthroplasty, shoulder surgery and spine surgery [21]. Our study found that patients, who are either female or at an older age, had generally higher expectations in hand surgery. As the impact of patient expectations on surgical outcome has been emphasized, it is important to manage patient expectations before surgery [22]. Identification of patient factors associated with preoperative expectations, as established by our analysis, will be useful in assisting surgeons to manage expectations at an appropriate level through patient education.

Several studies have confirmed a strong association between preoperative expectations and postoperative satisfaction in orthopaedic surgery [21]. In general, surgeons considered patients with high preoperative expectations tend to be more easily dissatisfied after surgery. In contrast, many studies have described that high expectations are related to positive surgical outcomes and patient satisfaction even if expectations have not been completely fulfilled [23]. Our study supports this hypothesis as shown by a positive relationship between preoperative expectations and postoperative satisfaction in patients who have undergone hand surgery. Patient satisfaction is a dynamic and multidimensional concept and is influenced by multiple factors including different diagnoses or procedures [24]. A study is needed to investigate how patient satisfaction varies depending on diagnoses and procedures in hand surgery. And, the survey we have designed can provide a framework for further research on this topic.

This study has some limitations. Firstly, patient population is not the same at Phases I and II in terms of diagnoses and demographic characteristics. Patients diagnosed with acute distal radius fracture were included fewer in Phase II than Phase I because the interval between their first and second outpatient visits for surgery was too short to be considered for the analysis of the test-retest reliability. However, despite the heterogeneity of the patient population, the test-retest reliability was high. Secondly, during the test-retest phase, the second interview was carried out in the hospital on the day of surgery. The psychological status of patients might have been affected while waiting for surgery, especially in the hospital where they are surrounded by other patients.

## Conclusion

Developing a valid and reliable survey questionnaire applicable to hand surgery in general is an essential prerequisite for understanding the impact of patient expectations. The survey we designed provides a simple way of identifying patient expectations and facilitates patient education with regard to expectation management through feedback. The study found that patients with high expectations tend to be more satisfied. It is strongly believed that this survey questionnaire would serve as a useful tool at a time when patient perspective is taken into account increasingly more in the clinical practice.

## Supporting information

S1 Fig Relationship between patient preoperative expectation scores and postoperative satisfaction scores. (TIF)

S2 Fig The original version of the questionnaire in Korean. (TIF)

S1 Table Extended comparison of demographic characteristics between phases I and II. (DOCX)

S2 Table Bivariate analysis of patient demographics in phase II. (DOCX)

## References

[1] Waljee JF, Chung KC. Commentary regarding "Evaluation of expectations and expectation fulfillment in patients treated for trapeziometacarpal osteoarthritis". J Hand Surg Am. 2015;40(3): 491–492. doi: 10.1016/j.jhsa.2014.11.014 25708435

[2] Frouzakis R, Herren DB, Marks M. Evaluation of expectations and expectation fulfillment in patients treated for trapeziometacarpal osteoarthritis. J Hand Surg Am. 2015;40(3): 483–490. doi: 10.1016/j.jhsa.2014.10.066 25617218

[3] Marks M, Herren DB, Vliet Vlieland TP, Simmen BR, Angst F, Goldhahn J. Determinants of patient satisfaction after orthopedic interventions to the hand: a review of the literature. Journal of hand therapy: official journal of the American Society of Hand Therapists. 2011;24(4): 303–312.e310; quiz 312. doi: 10.1016/j.jht.2011.04.004 21684112

[4] Zywiel MG, Mahomed A, Gandhi R, Perruccio AV, Mahomed NN. Measuring expectations in orthopaedic surgery: a systematic review. Clin Orthop Relat Res. 2013;471(11): 3446–3456. doi: 10.1007/s11999-013-3013-8 23633186 PMC3792280

[5] Cody EA, Mancuso CA, MacMahon A, Marinescu A, Burket JC, Drakos MC, et al. Development of an Expectations Survey for Patients Undergoing Foot and Ankle Surgery. Foot Ankle Int. 2016;37(12): 1277–1284. doi: 10.1177/1071100716666260 27654045

[6] Mancuso CA, Duculan R, Stal M, Girardi FP. Patients’ expectations of lumbar spine surgery. European spine journal: official publication of the European Spine Society, the European Spinal Deformity Society, and the European Section of the Cervical Spine Research Society. 2015;24(11): 2362–2369. doi: 10.1007/s00586-014-3597-z 25291976

[7] Mancuso CA, Altchek DW, Craig EV, Jones EC, Robbins L, Warren RF, et al. Patients’ expectations of shoulder surgery. J Shoulder Elbow Surg. 2002;11(6): 541–549. doi: 10.1067/mse.2002.126764 12469077

[8] Mancuso CA, Salvati EA, Johanson NA, Peterson MGE, Charlson ME. Patients’ expectations and satisfaction with total hip arthroplasty. The Journal of Arthroplasty. 1997;12(4): 387–396. doi: 10.1016/s0883-5403(97)90194-7 9195314

[9] Mancuso CA, Duculan R, Stal M, Girardi FP. Patients’ expectations of cervical spine surgery. Spine (Phila Pa 1976). 2014;39(14): 1157–1162. doi: 10.1097/BRS.0000000000000349 24732846

[10] Mancuso CA, Sculco TP, Wickiewicz TL, Jones EC, Robbins L, Warren RF, et al. Patients’ expectations of knee surgery. The Journal of bone and joint surgery American volume. 2001;83-a(7): 1005–1012. doi: 10.2106/00004623-200107000-00005 11451969

[11] Kim JK, Lim HM. The Korean version of the Carpal Tunnel Questionnaire. Cross cultural adaptation, reliability, validity and responsiveness. J Hand Surg Eur Vol. 2015;40(2): 200–205. doi: 10.1177/1753193414540083 25005562

[12] Kadzielski J, Malhotra LR, Zurakowski D, Lee SG, Jupiter JB, Ring D. Evaluation of preoperative expectations and patient satisfaction after carpal tunnel release. J Hand Surg Am. 2008;33(10): 1783–1788. doi: 10.1016/j.jhsa.2008.06.019 19084178

[13] Kang L, Hashmi SZ, Nguyen J, Lee SK, Weiland AJ, Mancuso CA. Patients With Thumb Carpometacarpal Arthritis Have Quantifiable Characteristic Expectations That Can Be Measured With a Survey. Clin Orthop Relat Res. 2016;474(1): 213–221. doi: 10.1007/s11999-015-4573-6 26443775 PMC4686505

[14] Kang L, Nguyen J, Hashmi SZ, Lee SK, Weiland AJ, Mancuso CA. What Demographic and Clinical Characteristics Correlate With Expectations With Trapeziometacarpal Arthritis? Clin Orthop Relat Res. 2017;475(11): 2704–2711. doi: 10.1007/s11999-017-5359-9 28425053 PMC5638728

[15] Ware JE Jr, Kosinski M, Keller SD. A 12-Item Short-Form Health Survey: construction of scales and preliminary tests of reliability and validity. Medical care. 1996. 220–233. doi: 10.1097/00005650-199603000-00003 8628042

[16] Iglesias C, Torgerson D. Does length of questionnaire matter? A randomised trial of response rates to a mailed questionnaire. Journal of health services research & policy. 2000;5(4): 219–221. 11184958 10.1177/135581960000500406

[17] O’Reilly-Shah VN. Factors influencing healthcare provider respondent fatigue answering a globally administered in-app survey. PeerJ. 2017;5: e3785. doi: 10.7717/peerj.3785 28924502 PMC5600176

[18] Dorman PJ, Slattery J, Farrell B, Dennis MS, Sandercock PA. A randomised comparison of the EuroQol and Short Form-36 after stroke. United Kingdom collaborators in the International Stroke Trial. BMJ (Clinical research ed). 1997;315(7106): 461. doi: 10.1136/bmj.315.7106.461 9284664 PMC2127345

[19] Saleh KJ, Bershadsky B, Cheng E, Kane R. Lessons Learned from the Hip and Knee Musculoskeletal Outcomes Data Evaluation and Management System. Clinical Orthopaedics and Related Research^®^. 2004;429: 272–278. doi: 10.1097/01.blo.0000137589.23853.61 15577498

[20] Johnson SP, Sebastin SJ, Rehim SA, Chung KC. The Importance of Hand Appearance as a Patient-Reported Outcome in Hand Surgery. Plastic and reconstructive surgery Global open. 2015;3(11): e552. doi: 10.1097/GOX.0000000000000550 26893977 PMC4727704

[21] Swarup I, Henn CM, Gulotta LV, Henn RF 3rd. Patient expectations and satisfaction in orthopaedic surgery: A review of the literature. Journal of clinical orthopaedics and trauma. 2019;10(4): 755–760. doi: 10.1016/j.jcot.2018.08.008 31316250 PMC6611830

[22] Mancuso CA, Graziano S, Briskie LM, Peterson MG, Pellicci PM, Salvati EA, et al. Randomized trials to modify patients’ preoperative expectations of hip and knee arthroplasties. Clin Orthop Relat Res. 2008;466(2): 424–431. doi: 10.1007/s11999-007-0052-z 18196427 PMC2505138

[23] Waljee J, McGlinn EP, Sears ED, Chung KC. Patient expectations and patient-reported outcomes in surgery: a systematic review. Surgery. 2014;155(5): 799–808. doi: 10.1016/j.surg.2013.12.015 24787107 PMC4170731

[24] Graham B. Defining and Measuring Patient Satisfaction. J Hand Surg Am. 2016;41(9): 929–931. doi: 10.1016/j.jhsa.2016.07.109 27570227

