# Supplementary figures and images for: Patient expectations and satisfaction in hand surgery: A new assessment approach through a valid and reliable survey questionnaire
Source: PLoS One. 2022 Dec 20;17(12):e0279341. doi: 10.1371/journal.pone.0279341 (PMC9767329; doi:10.1371/journal.pone.0279341)

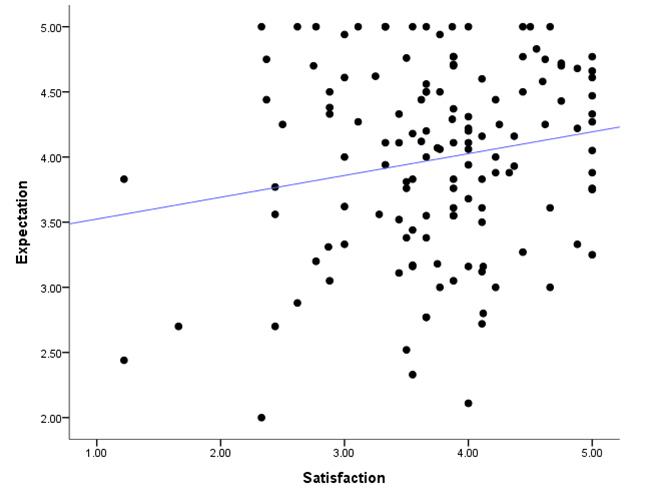

Supplement: S1 Fig — (TIF) [file pone.0279341.s001.tif]

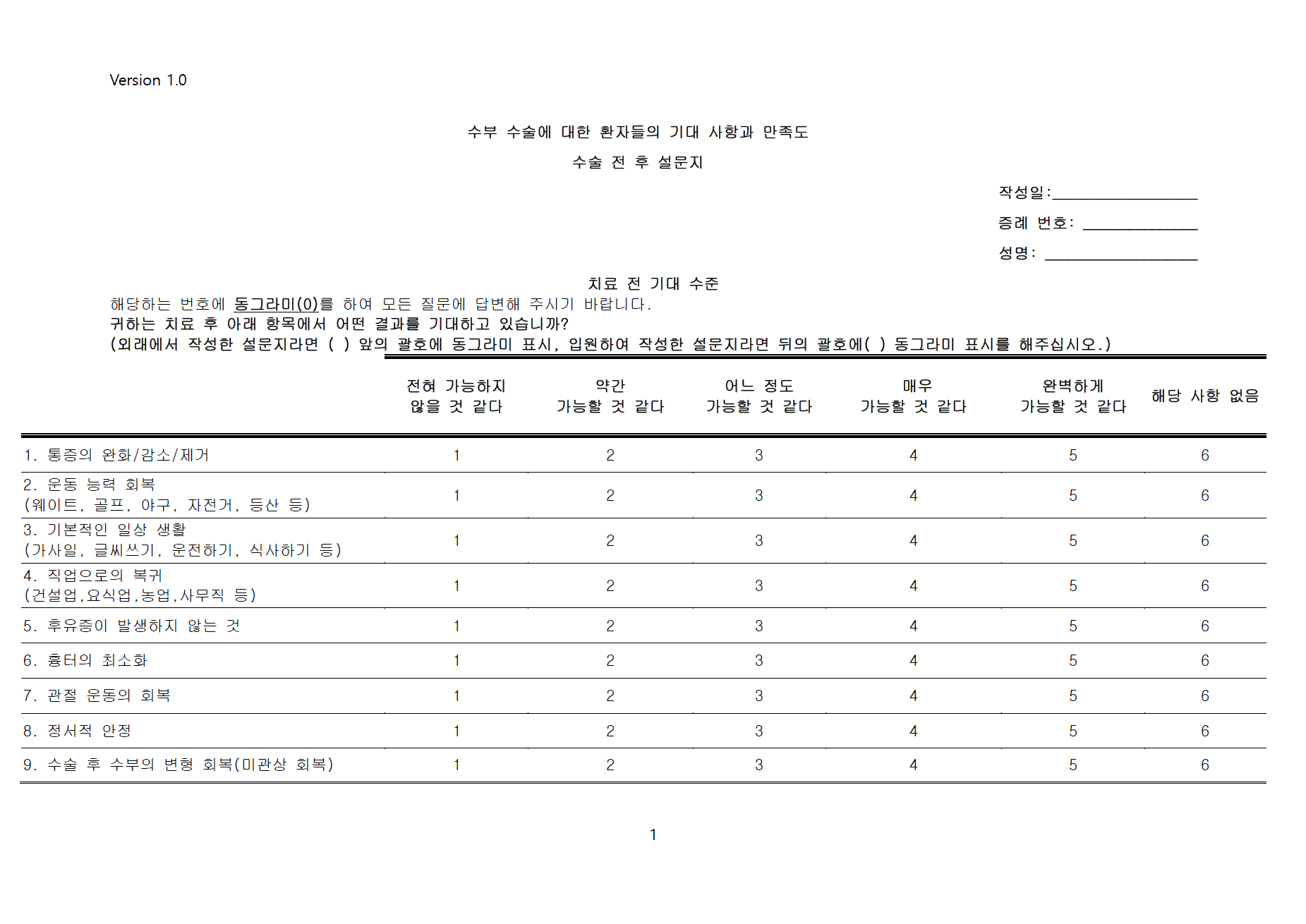

Supplement: S2 Fig — (TIF) [file pone.0279341.s002.tif]
