# Supplementary material for: Patient expectations and satisfaction in hand surgery: A new assessment approach through a valid and reliable survey questionnaire
Source: PLoS One. 2022 Dec 20;17(12):e0279341. doi: 10.1371/journal.pone.0279341 (PMC9767329; doi:10.1371/journal.pone.0279341)
Supplement: S1 Table — (DOCX) [file pone.0279341.s003.docx]

|  | Phase 1 | Phase 2 | p-Value |
| --- | --- | --- | --- |
| Average age, years, mean (SD) | 48 (16.03) | 51 (16.08) | 0.080 |
| Sex |  |  |  |
| Female | 59 (40.4%) | 83 (53.9%) | 0.019* |
| Male | 87 (59.6%) | 71 (46.1%) |  |
| Marital status |  |  |  |
| Married | 103 (70.5%) | 125 (81.2%) | 0.031* |
| Not married/divorced | 43 (29.5%) | 29 (18.8%) |  |
| Working status |  |  |  |
| Active | 90 (61.6%) | 97 (63%) | 0.810 |
| Inactive/retired | 56 (38.4%) | 57 (37%) |  |

S1 Table. Extended comparison of demographic characteristics between phases 1 and 2

SD, standard deviation.

*p < 0.05.
