# Supplementary material for: Patient expectations and satisfaction in hand surgery: A new assessment approach through a valid and reliable survey questionnaire
Source: PLoS One. 2022 Dec 20;17(12):e0279341. doi: 10.1371/journal.pone.0279341 (PMC9767329; doi:10.1371/journal.pone.0279341)
Supplement: S2 Table — (DOCX) [file pone.0279341.s004.docx]

| Demographic | Number  (SD or percentage) | Mean expectation score (SD) | p-Value |
| --- | --- | --- | --- |
| Average age (years) | 51 (16.08) |  |  |
| Sex |  |  |  |
| Male | 71 (46%) | 3.76 (0.68) |  |
| Female | 83 (54%) | 4.02 (0.66) | 0.022* |
| Marital status |  |  |  |
| Married | 125 (81%) | 3.99 (0.69) |  |
| Not married | 29 (19%) | 3.50 (0.51) | 0.001* |
| Working status |  |  |  |
| Active | 97 (63%) | 3.81 (0.67) |  |
| Inactive/retired | 57 (37%) | 4.04 (0.68) | 0.047* |
| Dominant hand affected? |  |  |  |
| Yes | 93 (60%) | 3.92 (0.68) |  |
| No | 61 (40%) | 3.87 (0.69) | 0.717 |
| History of hand injury or fracture |  |  |  |
| Yes | 59 (38%) | 3.85 (0.80) |  |
| No | 95 (62%) | 4.05 (0.68) | 0.133 |

S2 Table. Bivariate analysis of patient demographics in phase 2

SD, standard deviation

*p < 0.05.
